# Books

**Published:** 1882-10

**Authors:** 


### Books.

---

Diseases of the Ear in Children. By Anton Von Troeltsch, M D., Professor in the University of Wurzburg. Translated by J. Orne Green, A. M., M. D. From *Gerhardt's Handbuch Der Kinderkrankheiten.* New York: William Wood & Co. 1882.

The contents of this valuable little book of 165 pages are divided into five chapters, which treat respectively of Diseases of the external ear—the auricle, meatus and drum-membrane. Diseases of the middle ear—tympanum, Eustachian tube and mastoid processes. Foreign bodies in the ear. The diseases of the labyrinth and Deaf-mutism.

In this unpretending little volume we have a concise and eminently practical treatise upon a class of diseases which frequently come under the cognizance of the general medical practitioner, and which, we fear, do not receive the careful study and attention which their immediate and remote importance demands.

The following explicit directions are given to be observed in the use of the nasal douche or irrigator—which is recommended for the treatment of the diseased conditions of the pharyngeal mucous membrane and which often cause or keep up the ear disease: 1. Never use plain water, but if nothing special is ordered, either a solution of salt or milk and water. 2. Always have the water lukewarm—77° to 90° F. 3. Never have the reservoir higher above the nostrils than one or one and a half feet. 4. Avoid swallowing and breathe quietly through the mouth during the operation, and with children occasionally interrupt the current. 5. Direct the stream directly backwards, never upwards.

Hypertrophied tonsils, it is claimed, frequently maintain an inflammation of the pharynx, by acting as foreign bodies and producing mechanical irritation and disturbances of circulation. They also interfere with the movements of the palate, and, by pressing its posterior surface upwards against the tubal orifices, narrow or even close those orifices. Their early removal, by the tonsilotome, is urged. The advantages of this simple operation, commonly observed, are improvement of voice and articulation, and of the position of the mouth and facial expression, deeper respiration and consequently a fuller chest development, richer blood and more vigorous constitution.

Proper hygienic management is declared to be a *sine qua non*: not only at home, but in the school room as well.

A sensible caution is sounded against too officious, instrumental interference with foreign bodies in the ear.

The book is neatly and securely bound, and printed in handsome style on excellent paper. We notice a rather more frequent occurrence of typographical errors than is customary in the publications of this house, but with defects on this line we entertain only feelings of genuine sympathy.

---

Labor Among Primitive Peoples, Showing the Development of the Obstetric Science of To-day, from the Natural and Instinctive Customs of all races, civilized and savage, past and present. By George J. Engelmann, A.M., M.D. With fifty-six illustrations. St. Louis: J. H. Chambers & Co., 1882. Pp 203. Price, cloth $2.00.

Dr. Engelmann has succeeded in presenting to the profession a most entertaining and instructive brochure; and the apprehensions which he professes to feel lest he should be the subject of keen criticism are entirely groundless, for the quality of the matter more than compensates any real or imaginary defect in the manner of its arrangement.

This ethnological study, as it is termed by the author, consists in the combination, within the compass of one small volume, of three papers previously published; one in the Transactions of the American Gynecological Society, for 1880, and the other two in medical journals at different times.

The five chapters of the book are devoted to posture; management of the third stage of labor; pregnancy, parturition and child-bed; massage and expression; and characteristic labor scenes among the yellow, black and red races.

The illustrations are abundant, and, while some of the cuts are rather rudely executed, they, no doubt, convey a very correct idea of the peculiar habits of the people which they are intended to represent.

The writer in discussing the various obstetric positions says, not until he had undertaken this work, and had begun the study of positions assumed by savage and civilized women during labor, did he understand that there was method in the instinctive movements of women in the last stage of labor. "I have seen them," he says, "toss about, and have sought to quiet them; I bade them have patience, and lie still upon their backs; but since entering upon this study I have learned to look upon their movements in a very different light."

He condemns the dorsal decubitus during the parturient act, as it is unfavorable to the efficient assistance of the abdominal muscles. The recumbent position is rarely assumed among the women of savage tribes, hence he argues that it is unnatural and maintains that it is undesirable. Dr. Henry F. Campbell, of Augusta, is quoted in support of this last proposition.

The writer concludes that of all positions the semi-recumbent is the most serviceable, and should be adopted as the obstetric position in all ordinary labor cases.

We are constrained to think that our author is in error when he assumes that the dorsal position is generally practiced in this country. We have been led to believe that the favorite parturient position was lying on the left side with the chest drawn forward and the thighs flexed upon the abdomen, which is, for all practical purposes, the inclined, semi-recumbent position so warmly commended by the writer.

---

THE INCIDENTAL EFFECTS OF DRUGS. A Pharmacological and Clinical Hand-book. By Dr. L. Lewin, of the University of Berlin. Translated by W. T. Alexander, M.D. New York: William Wood & Co. 1882. Pp. 240.

In the author's preface we find the following paragraph, which explains the intent and scope of this work:

"But in the therapeutic employment of certain drugs, deviations sometimes occur from this typical and, as some may say, normal action, whose recognition and correct interpretation are not always easy. A knowledge of them is, however, of great importance to the physician, since they may, in a given case, shed light upon the cause of the unexpected phenomena which show themselves and furnish indications to guide him in his practical interference."

The translator, recognizing the importance of a knowledge of the incidental, accidental or unexpected effects of drugs upon the various organs and systems of the body, has made this interesting manual of Dr. Lewin available to the profession in America, and it is presented in a style characteristic of the enterprising house from which it emanates.

There can be no doubt concerning the practical importance of the field which this book seeks, in part, to occupy. It deserves to be largely read; and it is hoped that it will serve as a guide and a stimulus to physicians, inducing them to note carefully the exceptional and extraordinary effects of ordinary remedies and to report the results of their observations. In this way the literature of the subject will be greatly augmented and may be made very valuable to medical practitioners.

We are pleased to notice that although the metric system is followed in the text, it is translated, so to speak, into the terms in common use among physicians and apothecaries.

---

THE CHANGE OF LIFE IN HEALTH AND DISEASE. A Clinical Treatise on the Diseases of the Ganglionic Nervous System Incidental to Women at the Decline of Life. By Edward John Tilt, M.D. Fourth Edition. Philadelphia: P. Blakiston, Son & Co. 1882. Pp. 185. Price—cloth, $1.25; paper, 75 cents.

In this volume the accomplished author has endeavored to group in systematic arrangement the varied and often obscure and misty phenomena which pertain to that important period in the history of woman known as the change or decline of life, the climacteric, etc. As this is the fourth edition of the work, it is scarcely necessary for us to remark the success which has attended the effort; and the large experience and well-known conservatism of the writer will command attention, and will insure confidence in the views which he herein so pleasingly presents to the consideration of the medical profession.

"It may be well," he says, "to remind the reader of the several milestones on the road that leads from the cradle to the grave; seven, fourteen and twenty-one are clearly written on the first part of it; forty-two, forty-nine, and sixty-three, though less deeply cut, are distinctly visible on those that mark the last part of the journey. These dates point to periods of the human life characterized by very important changes, many of which give a peculiar aspect to the physiology of the human being, and impart a family likeness to the diseases of epochs justly deemed critical. No division of labor is so praiseworthy as the study of these great periods in human life; and if many of those who fritter away their meritorious energy in writing monographs, would devote it to thoroughly investigating the physiology and diseases of the great periods on which nature has put a special stamp, they would very much assist the young medical men who have to begin practice with a serviceable knowledge of such definite diseases as are gathered into hospitals and can be read of in books. They soon find, however, that such diseases are comparatively rare, and that their time is taken up in attending to a host of odd ailments they have never heard of, and of slight but complicated infirmities of various organs that find no place in medical literature."

The most available therapeutical resources to overcome the disorders described in this treatise are sedatives and tonics. Notably the bromides, hyoscyamus, chloral, camphor, etc., and nux vomica, quinine, arsenic, iron, etc. Of course, any specific uterine lesion may demand, as at other times, appropriate local treatment.

It is an entertaining and instructive little book, and while we, without hesitation, commend it to our readers, we can but wish that in his published writings, at least, the author would be a little more charitable, if not a little more just, toward his colaborers on this side of the Atlantic. Such a course would not detract from the value of his work at home or abroad, while it would, we feel confident, greatly increase respect for his opinions on both hemispheres.

MEDICAL COMMUNICATION OF THE MASSACHUSETTS MEDICAL SOCIETY. Vol. XIII. No. 1, 1882. Alfred Hosmer, M.D., Watertown, President. Francis W. Goss, M.D., Roxbury, Secretary.

This volume of transactions contains the proceedings of the councillors at a stated, an adjourned and an annual meeting, covering a period extending from October, 1881, to June, 1882, and several interesting articles. The treasurer's report shows that the income of the society last year was $9,192.49, and the expense account was $8,482.44, leaving a net balance in the treasury of $710.05.

One hundred and two new members were added to the society during the year.

The published papers are as follows: Infantile mortality; its causes and prevention, by James P. Linde, M.D., Some Obscure Mental Symptoms in Disease, by Charles F. Folsom, M.D., Relation of Mould Fungi to Disease, by William W. Gannett, M.D., A Study of the Action of Iron, by Francis H. Williams, M.D.

---

THE MULTUM IN PARVO REFERENCE AND DOSE BOOK. By C. Henri Leonard. M.A., M.D. Price Popular Edition, Paper 30, Cloth, 75 cents.

This little pamphlet represents very fairly what its title implies. Besides a list of old and new drugs with proper doses for different ages, it contains an obstetric calendar, directions for prescription writing, pelvic and fœtal measurements, rules for pronunciation, poisons and antidotes, tests for urinary deposits and other scientific and useful knick-knack bits of information.

